# Development of knowledge, attitude and practice questionnaires on e-Huffaz ProHealth, a multicomponent lifestyle intervention module among Tahfiz students

**DOI:** 10.1371/journal.pone.0309942

**Published:** 2024-09-26

**Authors:** Wan Nor Atikah Che Wan Mohd Rozali, Ismarulyusda Ishak, Arimi Fitri Mat Ludin, Amanina Athirah Mad Azli, Nurul ‘Izzah Solah, Farah Wahida Ibrahim, Nor Malia Abd Warif

**Affiliations:** 1 Faculty of Health Sciences, Biomedical Science Program, Universiti Kebangsaan Malaysia, Kuala Lumpur, Malaysia; 2 Faculty of Health Sciences, Center for Toxicology & Health Risk Studies (CORE), Universiti Kebangsaan Malaysia, Kuala Lumpur, Malaysia; 3 Faculty of Health Sciences, Center for Healthy Ageing & Wellness (H-CARE), Universiti Kebangsaan Malaysia, Kuala Lumpur, Malaysia; Universiti Monash Malaysia: Monash University Malaysia, MALAYSIA

## Abstract

e-Huffaz ProHealth is a lifestyle intervention module developed specifically for Tahfiz students. The e-Huffaz ProHealth consists of physical health, nutritional and psychological components. This study aimed to develop the knowledge, attitude and practice (KAP) questionnaires on e-Huffaz ProHealth among Tahfiz students. This cross-sectional pilot study was conducted in 2022. The first phase entailed developing the KAP questionnaires. The second phase involved determining the content validity and face validity. While the third phase involved evaluating the reliability. A total of six experts from public academic institutions participated in the initial evaluation stage to assess validity while five experts were involved in the second stage. Thirty-three and 41 respondents were involved for the face validity and reliability evaluation, respectively. The questionnaires were distributed via Google Docs and hard copies were handed out in person to Tahfiz school teachers and students at Madrasah Tahfiz Al-Amani and Pondok Moden Tahfiz Saadah Addaarain. The findings demonstrated that the Item Content Validity Index (I-CVI) for the three components of the KAP questionnaires at the second stage of evaluation was high (1.0). The scores for the Scale Face Validity Average Index (S-FVI/Average) in assessing the level of clarity and understanding for the three components were 0.89 and 0.88, 0.92 and 0.90, and 0.88 and 0.9, respectively. Meanwhile, the reliability of KAP for physical activity was moderate (0.43), very high (0.91) and high (0.7), respectively. For nutrition, the reliability of KAP was good and acceptable with the values of 0.63, 0.83 and 0.65, respectively. The results of reliability of KAP for psychological well-being was good with the values of 0.54, 0.56 and 0.84, respectively. The KAP questionnaires of e-Huffaz ProHealth was successfully developed with high content validity, good face validity and acceptable reliability. Hence, it can be used for future study to evaluate the effectiveness of e-Huffaz ProHealth among Tahfiz students.

## Introduction

e-Huffaz ProHealth is a lifestyle intervention module for Tahfiz school students developed by researchers from the Universiti Kebangsaan Malaysia Kuala Lumpur (UKMKL) in collaboration with the management group of the Al-Quran Tahfiz Institution Association of Selangor (PITAS). The module serves to guide Tahfiz students in achieving good physical and psychological well-being that includes aspects pertaining to physical health and psychological well-being, and balanced nutrition. The Tahfiz education system in Malaysia is rapidly evolving [1]. The physical, social and mental health of students can be affected due to the neglection of health aspects in Tahfiz schools, hampering the effort in ensuring the student’s well-being. Proper health habits and practices can help to enhance the health and quality of life for these students. According to the World Health Organization (WHO) [2], health is not merely the absence of disease (which is physical health), but in broader term, it refers to the three components: physical, mental and social health.

Research on 116 students from three Tahfiz schools in Selangor showed that the quality of life related to the physical health component was good and they spent 268 minutes a week on leisure activities [3]. However, this amount of time does not meet the recommendations of the WHO for the duration of leisure activities for teenagers, which is 60 minutes a day or 420 minutes a week. In addition, Tahfiz students were found to have high cholesterol levels [4]. This physical inactivity may be one of the causes of high cholesterol among students [5]. The needs assessment for physical activity found that 81% of students stated that playing sports or exercising affected their memory level because such activities could relieve stress and keep the body healthy and brain smart.

Balanced nutrient intake by Tahfiz students was less than the suggested daily nutrients because most Tahfiz schools were not able to provide the recommended healthy nutrition. The calories intake of Tahfiz students in Selangor is only 1015–1382 kcal/day. However, this number does not meet the Malaysian Nutritional Intake recommendation (RNI), which is 2210–2340 kcal/day. The consumption and practice of healthy eating among Tahfiz students are very important because they can contribute to the process of memorizing the Quran as well as improving the intelligence of their mind and body health [6]. Analysis of the student needs survey found that 90% of students were aware of the importance of healthy eating for health, but there were still many students who did not understand the meaning and concept of healthy eating. This shows that they do not have basic knowledge about the meaning and concept of proper healthy eating. In addition, the results of the analysis of aspects of healthy eating practices found that only 20% of Tahfiz students practiced healthy eating in their daily lives. In addition, as many as 58.3% of Tahfiz teachers responded that their students did not practice healthy food intake [7].

The needs study found that all teachers and 65% of students stated that emotions and calmness affected the level of memorization of students. Teachers believe that unstable emotions will make the memorization process difficult while good emotions will facilitate memorization due to the high enthusiasm. In addition, the teacher also stated that memorizing the Quran required high mental strength. According to the students, they could not memorize the Quran if they were stressed and sad. They also responded that old memories could be lost if they experienced stress [8].

Physical health can be defined as a state of an individual being free from pain, physical disability, chronic and infectious disease and bodily discomfort that necessitates medical attention [9]. Nutritional status is a state of an individual as a result of nutrition intake, absorption and utilization [10]. Psychological well-being entails being satisfied with one’s life and understanding the positive emotions [11]. Each component has its own subunit that serves as a guideline for students who will participate in the intervention.

The effectiveness of e-huffaz ProHealth not yet been tested. To achieve this, a set of questionnaires to assess the knowledge, attitude and practice (KAP) of students towards their physical, nutritional and psychological well-being was developed to evaluate the effectiveness of the module. The KAP questionnaire is widely used in the field of health science as it is simple and easy to develop and implement, and it provides extensive information and does not require a large cost [12]. The KAP model is developed as a tool to evaluate what the sample knows, believes and acts about a certain topic [13]. Each study that uses the KAP model has a different scoring system. The type of measurement scale and the number of items used can vary depending on the appropriateness of the study [14]. Taken together, the current study aimed to develop a KAP-based questionnaire on e-Huffaz ProHealth, which would be a multi-component lifestyle intervention module among Tahfiz students. The findings of the validity and reliability of this KAP questionnaires can be served as a guideline for other researchers to conduct similar multi-dimensional health intervention study in the future.

## Materials and methods

### Study designs and setting

Our aim was to develop a set of KAP questionnaires on e-Huffaz ProHealth in Malay language.This e-Huffaz ProHealth is a multi-component lifestyle intervention module on Tahfiz students. This newly developed questionnaire was piloted among experts from the higher education institutions as well as the teachers and students from Madrasah Tahfiz Al-Amani and Pondok Moden Tahfiz Saadah Addaarain. The recruitment period was between 01 Jun 2022 to 30 Jun 2022. This study was approved by the Human Research Ethics Committee Universiti Kebangsaan Malaysia, JEP-2022-229 prior to conducting the study. All participants provided their written informed consent prior to the study. Whereas the parental written informed consent was obtained for the underage students. The personal identifiers of all participants were kept confidential.

**Fig 1:**
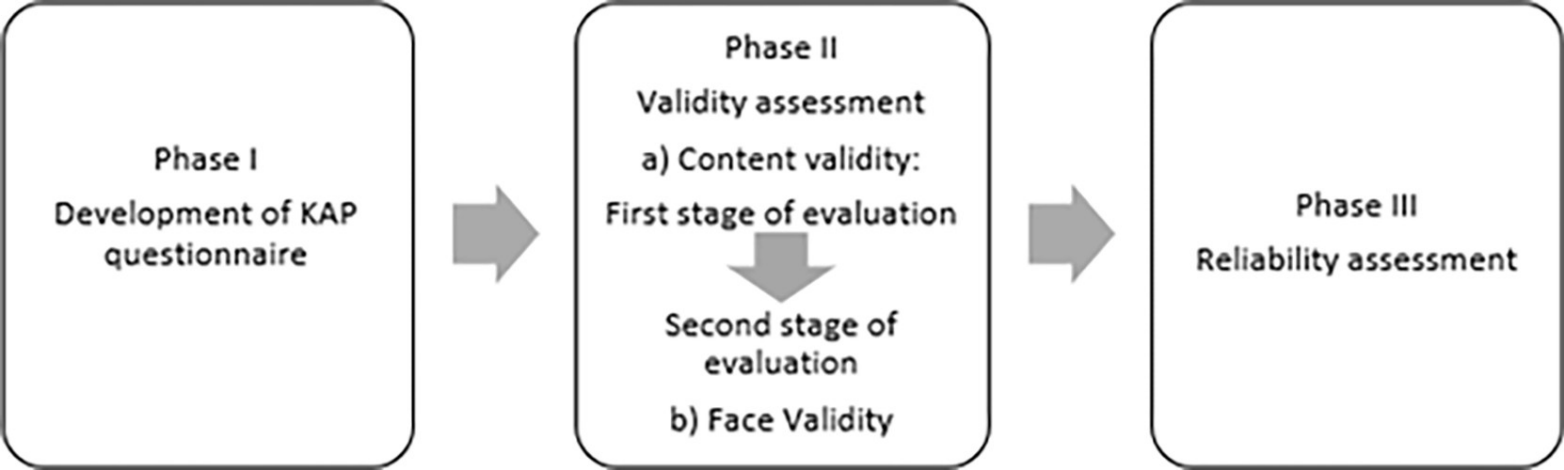
Workflow of the phases involved.

This study consisted of three phases: phase I: KAP questionnaires development; phase II: content validity and face validity; and phase III: reliability assessment. The workflow of the phases involved is as depicted in Fig 1.

### Study participants and sampling

In the first stage of Phase II, there were six experts from four public academic institutions involved in the content validity assessment. These were the experts from Universiti Kebangsaan Malaysia (UKM), Universiti Pendidikan Sultan Idris (UPSI), Universiti Sains Islam Malaysia (USIM) and Universiti Sains Malaysia (USM). The selection of the experts was based on their expertise in relation to the objectives of all the components of e-Huffaz ProHealth. In the second stage of Phase II, the KAP questionnaires were distributed to two private Tahfiz schools in Selangor to assess the face validity and reliability of the module. This assessment involved the Tahfiz school teachers and students at Madrasah Tahfiz Al-Amani and Pondok Moden Tahfiz Saadah Addaarain. Participants were selected through purposive sampling. Teachers meeting the criteria of being aged 18 and above, having responsibility for boarding students, possessing over 6 months of teaching experience, and having the ability to comprehend the Malay language were included. As for students they needed to be aged between 13 and 17 years, residing in the hostel, and having proficiency in the Malay language. However, individuals diagnosed with any mental illnesses were not included in the study.

The sample size for the main study for teachers and students was calculated using G*Power 3.1.9.7. The determination of the sample size for teachers relies on correlation analysis within the Cronbach’s alpha reliability test. A substantial effect size of 0.3 [15] and when factoring in a 50% attrition rate resulted in a sample size of 123. While for students, considering *F*-test family, with an effect size of *f* = 0.095 [16], α = 0.05, 1 –β = 0.9, number of groups = 2, number of measurements = 3, correlation among repeated measures = 0.5, € = 1, resulted in a sample size of 180. With a dropout rate of 50%, the final sample size is 270 students. According to Connelly (2008), the sample size for pilot studies should be calculated as 10% of the main study’s total [17]. Therefore, there were approximately 12 teachers and 27 students involved in this pilot study.

## Data collection and tool and technique

### Phase I

#### KAP questionnaires development

The KAP questionnaires of e-Huffaz ProHealth, a multi-component lifestyle intervention module for Tahfiz students, was developed based on the Healthy Lifestyle Program Module [18–20] and the objective of each component in the module itself. The KAP questionnaire was also constructed based on a previously performed needs analysis [21]. Several objectives that represented the three main components in e-Huffaz ProHealth Module were used as the reference to construct the items in the KAP questionnaires [21]. After a series of team discussion, a comprehensive KAP questionnaires encompassing physical health, nutrition, and psychological well-being components were successfully developed. All questions were in close-ended form. The questions on attitude and practice domain were based on Likert scale. For the knowledge domain, the answers should be between ‘yes’, ‘no’ or ‘don’t know’. For attitude domain, 5-point Likert scale was used: ‘1 = Strongly disagree’, ‘2 = Disagree’, ‘3 = Not sure’, ‘4 = Agree’ and ‘5 = strongly agree’. For the practice domain, 4-point Likert scale was used: ‘1 = Often’, ‘2 = Sometimes’, ‘3 = Rarely’ and ‘4 = Not at all’.

### Phase II

#### Content validity

The content validity evaluation was carried out online to a panel of six experts. All expert panels selected are based on their field of expertise, level of education, and experience relevant to the components of the constructed questionnaire. They consist of academicians from higher education institutions of Universiti Kebangsaan Malaysia (UKM), Universiti Pendidikan Sultan Idris (UPSI), Universiti Sains Islam Malaysia (USIM), and Universiti Sains Malaysia (USM). These panels specialize in the fields of exercise physiology and sport science, nutritional science, biomedical science, clinical psychology and behavioral science. In the content validity questionnaires, the division of questions, instructions and steps to evaluate the questionnaires were clearly provided. At the first stage of evaluation, the panels were asked to critically evaluate the objectives, domains and items of the KAP questionnaires. The panels were required to score each item in the evaluation form. They were also advised to provide comments on items that needed improvement. The items in the KAP questionnaires were then amended based on the comments received from the panels. Next, the questionnaires were re-evaluated by the same panels for the second stage of evaluation. The evaluation process was done according to the procedures previously described [22].

The accepted content validity index (CVI) value from at least six panel experts was 0.83 [23]. The calculation of the CVI content validity value was done after getting the evaluation results from the six experts. In the CVI calculation, the relevance score should be recorded as one (relevance score of three or four) or zero (relevance score of one or two). Meanwhile, the content validity was determined through the item-content validity index (I-CVI) values. A good content validity value can be achieved when having an I-CVI value of at least 0.83.

Scale-level content validity with an average index (S-CVI/Ave) shows the content validity index scale value based on the average method. Meanwhile, scale-level content validity with a universal agreement index (S-CVI/UA) shows the value of the content validity index scale based on the universal panel agreement method. The minimum S-CVI/UA level of 0.80 indicates an excellent content validity [22]. The content validity values were calculated based on the formula by Yusoff (2019) [24].

#### Face validity

The face validity assessment was measured using a Likert scale score for the level of clarity (1-Item is not clear; 2-Item is somewhat clear; 3-Item is clear; 4-Item is very clear) and the level of understanding (1-Item is not comprehensible; 2-Item somewhat comprehensible; 3-Item comprehensible; 4-Item very comprehensible). The face validity assessment was analysed using the items-face validity index (I-FVI) and scale-level face validity with an average index (S-FVI/Ave) based on the formula by Yusoff (2019) [24].

### Phase III

#### Reliability

The reliability of the questionnaires was assessed by using two analysis methods: Kuder-Richardson Formula 20 (KR-20) analysis and Cronbach Alpha analysis. The KR-20 analysis was used to assess the knowledge domain because the questionnaires items used dichotomous response options [23]. Meanwhile, Cronbach Alpha analysis was used to assess the attitude and practice domains because the questionnaires items used multiple choice in the form of Likert scale scores.

## Results

### Phase I

#### KAP questionnaires development

There were four main parts in the KAP questionnaires of e-Huffaz ProHealth, which comprised of demographics data, physical health, nutritional and psychological well-being components. All components in the KAP questionnaires were divided into three main domains, namely knowledge, attitude and practice. Each domain for the physical health component consisted of 10 items. Meanwhile, each domain for nutritional and psychological well-being component consisted of 9 items and 10 items, respectively. Table 1 shows the summary of domains and components of the KAP questionnaires.

**Table 1:** Summary of domains and components of the KAP questionnaire.

Components	Domains	No. of items	Measurements	Response choices
Physical Health	Knowledge	10 (K1-K10)	Personal hygiene, hand washing steps, exercise, body posture, aedes and leptospirosis.	Yes/No/Don’t know
	Attitude	10 (A1-A10)	Flushing the toilet, gotong-royong, use of soap, sedentary life, body stretching, physical exercise, sweeping the floor and disposing of stagnant water.	1 = Strongly disagree 2 = Disagree 3 = Not sure 4 = Agree 5 = Strongly agree
	Practice	10 (P1-P10)	Wash hands, stretching activities, exercise every day, sit in a posture, cover the food and clean clothes.	1 = Often 2 = Sometimes 3 = Rarely 4 = Not at all
Nutritional	Knowledge	9 (K1-K9)	Food pyramid, half quarters concept, balanced diet, fried foods, vegetables consumption, carbohydrate intake, plain water, cooked food, recognize spoiled food.	Yes/No/Don’t know
	Attitude	9 (A1-A9)	Prioritize balanced food, eat fruit, eating breakfast, low sugar content food, steamed or grilled food, high salt and flavouring content food, amount of rice, quantity of vegetables, check the expiration date.	1 = Strongly disagree 2 = Disagree 3 = Not sure 4 = Agree 5 = Strongly agree
	Practice	9 (P1-P9)	Food pyramid, eat fruit, safety of food, half-quarter concept, food >4 hours, fatty foods, fast food and high sugar content.	1 = Often 2 = Sometimes 3 = Rarely 4 = Not at all
Psychological well-being	Knowledge	10 (K1-K10)	Self-motivation, time management, short-term stress, sharing problems, effective communication, memorizing of Quran.	Yes/No/Don’t know
	Attitude	10 (A1-A10)	Help friends, respect the privacy, care, work in a team, listen to Quran, express problems, to-do lists, schedule and self-reward.	1 = Strongly disagree 2 = Disagree 3 = Not sure 4 = Agree 5 = Strongly agree
	Practice	10 (P1-P10)	Time management, ask for help, favorite activities, lend a helping hand, polite tone, ask for prayers, express problems, see a counsellor, deep breathe techniques and investigate the news.	1 = Often 2 = Sometimes 3 = Rarely 4 = Not at all

### Phase II

#### Content validity

*First stage of evaluation*. The following results show the first stage of the evaluation of content validity. The I-CVI values of the KAP questionnaires on e-Huffaz ProHealth for the multi-component lifestyle are shown in Appendix I. Table 2 shows the S-CVI/Ave and S-CVI/UA values.

**Table 2:** S-CVI/Ave and S-CVI/UA values for the first stage of evaluation.

Component	Domain	S-CVI/Ave	S-CVI/UA
Physical health	Knowledge	0.94	0.83
	Attitude	0.76	0.1
	Practice	0.95	0.71
	Overall	0.88	0.55
Nutritional	Knowledge	0.94	0.67
	Attitude	1	1
	Practice	0.93	0.6
	Overall	0.96	0.76
Psychological well-being	Knowledge	0.89	0.5
	Attitude	0.81	0
	Practice	0.94	0.78
	Overall	0.88	0.43

*Second stage of evaluation*. The second evaluation was carried out for the purpose of reassessing the validity of the content after the questionnaire’s items were improved based on feedback obtained in order to obtain a higher value of content validity. After the improvement, the KAP questionnaires for the physical health component contained a total of 30 items, with 10 items for each domain. The nutritional and psychological well-being had 27 items (9 items for each domain) and 30 items (10 items for each domain), respectively. The results for I-CVI, S-CVI/ave and S-CVI/UA for all the components were 1.

#### Face validity

The face validity assessment was done on 41 respondents. Table 3 shows the demographic data of respondents involved in this study. Table 4 shows the S-FVI/Ave values for all components. This face validity assessment is to measure the level of clarity and the level of understanding of the respondents regarding the KAP questionnaires.

**Table 3:** Demographic data for face validity and reliability assessment.

	Categories	n (%)
Sex	Male	18 (43.9%)
	Female	23 (56.1%)
Status	Teacher (22–40 years)	11 (26.8%)
	Student (13–17 years)	30 (73.2%)
Location	Madrasah Tahfiz Al-Amani, Banting, Selangor	17 (41.5%)
	Pondok Moden Tahfiz Saadah Addaarain, Teluk Panglima Garang, Selangor	24 (58.5%)

**Table 4:** S-FVI/Ave and S-FVI/UA values for all components.

Component	Domain	S-FVI/Ave	
		Clarity	Understanding
Physical health (n = 41)	Knowledge	0.84	0.85
	Attitude	0.93	0.91
	Practice	0.90	0.88
	Overall	0.89	0.88
Nutritional (n = 41)	Knowledge	0.92	0.88
	Attitude	0.90	0.90
	Practice	0.93	0.92
	Overall	0.92	0.90
Psychological well-being (n = 41)	Knowledge	0.90	0.88
	Attitude	0.91	0.90
	Practice	0.88	0.90
	Overall	0.90	0.89

### Phase III

#### Reliability

The reliability assessment was also done on 41 respondents. For the knowledge part, the data analysis was done using the Kuder-Richardson (KR-20) formula while the data analysis on the attitude and practice was measured using Cronbach Alpha. Table 5 shows the results of reliability assessment.

**Table 5:** Reliability assessment.

Component	Domain	No. of items	Reliability	Scores	Interpretation
Physical health (n = 41)	Knowledge	10	KR-20	0.43	Moderate
	Attitude	10	Cronbach Alpha	0.91	Very high
	Practice	10	Cronbach Alpha	0.70	Moderate
Nutritional (n = 41)	Knowledge	9	KR-20	0.63	Moderate
	Attitude	9	Cronbach Alpha	0.83	High
	Practice	9	Cronbach Alpha	0.65	Moderate
Psychological Well-being (n = 41)	Knowledge	10	KR-20	0.54	Moderate
	Attitude	10	Cronbach Alpha	0.56	Moderate
	Practice	10	Cronbach Alpha	0.84	High

## Discussion

In this study, the KAP questionnaires have been successfully developed and obtained good validity. The development of the questionnaires items based on the components and objectives of e-Huffaz ProHealth is important to obtain results that specifically measure the effectiveness of the module. The KAP questionnaires were often used for health-related interventions [23]. Moreira et al. [25] have used the KAP questionnaires to examine the effectiveness of an educational intervention on 82 adults in Brazil. This shows that KAP questionnaires are relevant to determine the effectiveness of a module.

The focus of this study was to develop a KAP questionnaires on e-Huffaz ProHealth, a multi-component lifestyle intervention module among Tahfiz students. The content validity, face validity and reliability of the developed questionnaires were evaluated. This was to identify the items that needed to be improved and the suitability of the items according to the objectives of the e-Huffaz ProHealth. If the KAP questionnaires did not have a good content validity, it would give the impression that the evaluation results did not measure what was intended to be measured and therefore, could not determine the effectiveness of the module. In the evaluation of content validity, a total of six experts of related fields evaluated and commented on the questionnaire’s items. This evaluation was done in two rounds because the first stage of evaluation resulted in CVI values of less than satisfactory as well as few constructive comments from the panel of experts to be considered. The amendments were made accordingly to obtain a good content validity of the questionnaires before proceeding to the next evaluation on the face validity and reliability.

The CVI value is the most widely used parameter in determining a quantitative evaluation [26]. The CVI value was used in measuring the content validity of KAP questionnaires [27]. At the first stage of evaluation, all items for the knowledge domain of physical health, nutritional and psychological well-being components were all good except for item K3 of physical health and item K4 of psychological well-being. Both items were suggested to be improved due to poor and unacceptable I-CVI values. According to the panel of experts, both items were unrelated to the objectives of e-Huffaz ProHealth and thus should be amended accordingly. In the second stage of evaluation, the I-CVI, S-CVI/Ave and S-CVI/UA values for all the components were good. The minimum CVI level accepted for six expert panels was 0.83 [22]. This means that the knowledge domain for all components was good and acceptable.

In the attitude domain, the first stage of the evaluation showed that the four items on the physical health component resulted in poor I-CVI values because the items (A1, A5, A9 and A10) were not addressing the attitude domain. All these items were then improvised to meet the criteria for the attitude domain. For the psychological well-being component, item K5 was criticized due to the improper sentences used in the questionnaires. Items that had poor I-CVI values needed to be revised [28]. However, items with very poor I-CVI values must be eliminated. Hence, four physical and one psychological well-being items were eliminated. At the second stage of evaluation, the I-CVI, S-CVI/Ave and S-CVI/UA values for all components were scored as good. This means that the attitude domain for all components was acceptable.

Next, in the practice domain, in the first stage of evaluation, only one item for psychological well-being needed to be revised as the question was not oriented towards motivation, but emphasised more on time and stress management. At the second stage of evaluation, the I-CVI, S-CVI/Ave and S-CVI/UA values for all components were good, meaning that the practice domain for all components was also acceptable. The S-CVI/Ave is a method that can overestimate content validity because the numerator will always be greater than the S-CVI/UA numerator if the I-CVI value is not all one [29]. For this reason, both S-CVI/UA and S-CVI/Ave were calculated, and the overall content validity might lie somewhere in between. The S-CVI/UA method likely underestimates the completeness of the questionnaires because it is calculated by adding all items with the equal I-CVI values to one divided by the total number of items [26].

Face validity is conducted to assess whether the questionnaires seem relevant or not in measuring a construct. Questions perceived as irrelevant will cause the respondents to either not answering honestly, transparently, or seriously. Therefore, face validity is conducted so that it appears relevant by testing the level of clarity and the level of understanding of the respondents on the questionnaire’s items. In this study, a total of 41 respondents were involved in the face validity assessment. The difference between face validity and content validity is that the content validity was assessed by experts from related fields whereas the face validity was assessed by respondents with the same population as the study [30]. This is because, the evaluation by the respondents would give feedback on whether they could understand the questionnaires items due to their age and lifestyle which might differ from the expert panels. Next, the researchers also played a role in ensuring that the questionnaires engaged appropriate use of language and were comprehensible to obtain good face validity.

The face validity of the questionnaires to assess the clarity and understanding is good when it exceeds 0.8 [31]. The face validity analysis of the KAP questionnaires showed that the S-FVI/ave values for the level of clarity and the level of understanding for all components were high and acceptable. These high values were obtained after the questionnaire’s items were re-evaluated. This was in line with the feedback from the panel of experts in the stage of refining the questionnaires, specifically at the second stage of content validity assessment. Therefore, improvements and amendments in terms of the use of appropriate phrases or sentences have been made based on the comments of the experts so that the understanding of the related questionnaires items can be improved. The S-FVI/UA values were not considered in this study because the face validity study only used S-FVI/Ave in evaluating the validity of the questionnaires. Therefore, the face validity of this KAP questionnaires were good and acceptable [32,33].

Reliability assessment was evaluated with two methods, the KR-20 method for the knowledge part and the Cronbach Alpha method for the attitude and practice part of the KAP questionnaires. Kuder-Richardson was used to measure the reliability of dichotomous questions. On the knowledge domain of the questionnaires, the KR-20 values for all components were moderate. Even though the values were acceptable, and the knowledge domain was considered as good before, KR-20 tended to produce more conservative estimations than Cronbach Alpha and improvement of questionnaires items should be done [34]. To increase the reliability of the questionnaires, the researcher recommended that items with two negative points (bisceral points) should be removed and that the number of items should be increased [35].

For the evaluation of the attitude and practice domain, Cronbach Alpha was used because the questions used Likert scale answers. The Cronbach Alpha values for the attitude domain for physical health and nutritional components were high. However, the psychological well-being component had a moderate Cronbach Alpha value. The minimum level of Cronbach Alpha reliability accepted is 0.7 [36]. This shows that the attitude domain for the physical health or well-being and nutritional component was acceptable because the closer the value was to one, the higher the internal consistency of the questionnaire’s items. Meanwhile, the improvement of questionnaires items should be done for the psychological well-being component.

The practice domain showed that only the psychological component had a high Cronbach Alpha value. Improvement of questionnaires items should be made for the physical health and nutritional component. One item was removed from the KAP questionnaires of physical health to increase the Cronbach Alpha. The attitude and practice section of the questionnaires had high reliability and therefore, they were accepted. The attitude and practice section questionnaires had high reliability, and good internal consistency value because it exceeded 0.7. An alpha index value of .50 was considered good for instrument scales that had less than ten items [37]. Therefore, the level of reliability for the KAP questionnaires of e-Huffaz ProHealth was considered good and acceptable.

## Limitation and recommendation

There are several limitations that have been identified through this study. The use of different questionnaire distribution methods between google form and self-distribution to both Tahfiz schools may affect the face validity assessment. This is because the level of clarity and understanding of instructions and guidance have different clarity. Additionally, a larger sample size and factor analysis must be considered for future studies to reduce the questionnaire items to specific constructs under the objective research and to achieve higher reliability.

## Conclusion

This study has successfully achieved good validity and reliability in the development of the KAP questionnaires on e-Huffaz ProHealth, a multi-component lifestyle intervention module among Tahfiz Students. This KAP questionnaires have been systematically developed based on the subunits in e-Huffaz ProHealth. Therefore, this KAP questionnaires are suitable to be used to measure the effectiveness of physical health, nutritional and psychological well-being components of e-Huffaz ProHealth in the field of the study. The items in this KAP questionnaires can also be served as a guideline for other researchers.

## Supporting information

S1 File Knowledge, attitude and practice (KAP) questionnaires on e-Huffaz ProHealth. (PDF)

S2 File Data collection. (PDF)
