# Supplementary material for: Development of knowledge, attitude and practice questionnaires on e-Huffaz ProHealth, a multicomponent lifestyle intervention module among Tahfiz students
Source: PLoS One. 2024 Sep 26;19(9):e0309942. doi: 10.1371/journal.pone.0309942 (PMC11426467; doi:10.1371/journal.pone.0309942)
Supplement: S1 File — (PDF) [file pone.0309942.s001.pdf]

Knowledge, attitude and practice (KAP) questionnaires on e-Huffaz ProHealth

This section contains statements related to physical health, nutritional and psychological well-being. Please read each statement and choose the best answer in the space provided.

For the **knowledge** domain, please circle only **ONE** answer between:
1= yes
2= no
3- don't know

For the **attitude domain**, please circle only **ONE** answer from the 5-point Likert scale below:

1= Strongly disagree
2= Disagree
3= Not sure
4= Agree
5= strongly agree

For the **practice** domain, please circle only **ONE** answer from the 4-point Likert scale below:
1= Often
2= Sometimes
3= Rarely
4= Not at all

**Physical Health**						
**Knowledge**						
K1	Personal hygiene can maintain human health.	1	2	3		
K2	Based on the recommendations of the Malaysian Ministry of Health, washing your hands properly requires following 5 hand washing steps.	1	2	3		
K3	Stagnant areas of clean water can cause breeding grounds for Aedes.	1	2	3		
K4	Diarrhea is a disease caused by not maintaining personal hygiene.	1	2	3		
K5	The World Health Organization (WHO) recommends a target time for each exercise of at least 30 minutes per day.	1	2	3		
K6	Once the exercise is done, one needs to cool down the body by slowing down the pace of movement to allow the body to enter the rest phase.	1	2	3		
K7	Exercising regularly can reduce the risk of heart attack.	1	2	3		
K8	The picture below shows the correct posture when sitting. 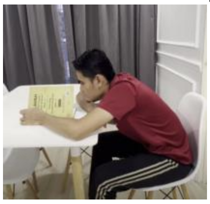	1	2	3		

K9	Leptospirosis disease is easily spread through the air.	1	2	3		
K10	Picking up items with the correct body posture is to avoid injury to the back of the body.	1	2	3		
**Attitude**						
A1	Personal hygiene must be always maintained.	1	2	3	4	5
A2	Flushing the toilet is necessary every time after use.	1	2	3	4	5
A3	Gotong-royong is an important activity that needs to be done regularly.	1	2	3	4	5
A4	The use of soap is important every time you wash your hands.	1	2	3	4	5
A5	A sedentary lifestyle should be avoided as much as possible.	1	2	3	4	5
A6	Cool down is important after exercise.	1	2	3	4	5
A7	Stretching should be done before starting every sporting activity.	1	2	3	4	5
A8	Physical exercise should be done even if there are no facilities at school.	1	2	3	4	5
A9	Sweeping the floor should be done every day.	1	2	3	4	5
A10	Disposing of stagnant water in the container should be done every week.	1	2	3	4	5
**Practice**						
P1	I wash my hands after I leave the toilet.	1	2	3	4	
P2	I wash my hands after contact with blood/pus/snot/saliva.	1	2	3	4	
P3	I wash my hands for at least 20 seconds.	1	2	3	4	
P4	I wash my hands by rubbing between the fingers as recommended by the Malaysian Ministry of Health.	1	2	3	4	
P5	I will do stretching activities at least 3 times a day.	1	2	3	4	
P6	I exercise every day for 30 minutes.	1	2	3	4	
P7	I sit in a posture that doesn't bend over.	1	2	3	4	
P8	I cover the food, so it is not exposed.	1	2	3	4	
P9	I exercise every day.	1	2	3	4	
P10	I always make sure my clothes are clean.	1	2	3	4	

**Nutritional**						
**Knowledge**						
K1	Consuming food according to the concept of the Malaysian Food Pyramid can maintain the health of the body.	1	2	3		
K2	The concept of half quarters as in the picture below needs to be practiced achieving a balanced and healthy diet. 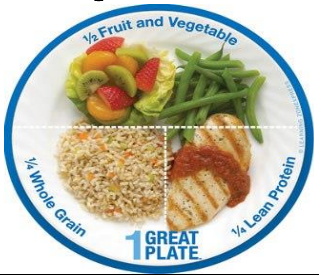	1	2	3		
K3	Eating a balanced diet means eating according to the guidelines of the food pyramid.	1	2	3		
K4	The intake of fatty foods can be reduced by limiting the intake of fried foods.	1	2	3		
K5	According to the Malaysian Food Pyramid 2020, the consumption of vegetables is the most consumption which is at least 3 servings of vegetables per day.	1	2	3		
K6	Excessive carbohydrate intake can increase the risk of obesity.	1	2	3		
K7	Drinking plain water is recommended to drink 2-3 glasses a day.	1	2	3		
K8	Cooked food can be stored for 2 days at room temperature.	1	2	3		
K9	The steps to recognize spoiled food are by looking, smelling and tasting.	1	2	3		
**Attitude**						
A1	I prioritize the selection of balanced food at every meal.	1	2	3	4	5
A2	I need to eat fruit every day.	1	2	3	4	5
A3	Eating breakfast is important to provide energy for daily activities.	1	2	3	4	5
A4	I prefer foods that have a low sugar content.	1	2	3	4	5
A5	I prefer steamed or grilled food over fried food.	1	2	3	4	5
A6	I like to eat foods that have a high salt and added flavoring content.	1	2	3	4	5
A7	I make sure the amount of rice is a quarter of the size of the food plate every meal.	1	2	3	4	5
A8	I make sure the quantity of vegetables is half the size of the food plate at every meal.	1	2	3	4	5
A9	I need to check the expiration date of the food before eating it.	1	2	3	4	5

Practice						
P1	I eat according to the recommendations of the Malaysian Food Pyramid.	1	2	3	4	
P2	I eat fruit every day.	1	2	3	4	
P3	When ordering food from outside, I make sure the food is safe to eat.	1	2	3	4	
P4	I eat according to the Half-Quarters concept.	1	2	3	4	
P5	I don't eat food that has been exposed and left for too long (>4 hours) at room temperature.	1	2	3	4	
P6	I see, taste and smell the food first before eating.	1	2	3	4	
P7	I eat the least amount of fatty foods compared to other food groups.	1	2	3	4	
P8	I like to eat fast food like burgers.	1	2	3	4	
P9	I don't eat foods that have a high sugar content.	1	2	3	4	

**Psychological well-being**						
**Knowledge**						
K1	Self-motivation is an internal drive to achieve life's goals.	1	2	3		
K2	Lack of self-motivation can lead to a decrease in quality of life.	1	2	3		
K3	Organized time management is important to increase one's productivity.	1	2	3		
K4	The Malaysian Ministry of Health recommends short-term stress control methods such as staying calm.	1	2	3		
K5	Sharing problems with someone you trust can increase your emotional burden.	1	2	3		
K6	Dominating the conversation is one of the characteristics of effective communication.	1	2	3		
K7	Learning about the stories of friends can increase self-motivation.	1	2	3		
K8	Memorizing the Quran can increase happy hormones (serotonin).	1	2	3		
K9	Repeating the memorization of the Quran can improve a person's memory.	1	2	3		
K10	Memorizing the Quran can increase a person's intellectual intelligence (IQ).	1	2	3		
**Attitude**						
A1	I like to help my friends when they are in trouble.	1	2	3	4	5
A2	I respect the privacy of others.	1	2	3	4	5
A3	I don't care when work is delayed.	1	2	3	4	5
A4	I work well in a team.	1	2	3	4	5
A5	I like to listen to the melody of the verses of the Quran when I'm stressed.	1	2	3	4	5
A6	I don't like to express my problems to someone when I am in trouble.	1	2	3	4	5
A7	I like to hurt myself when I'm stressed.	1	2	3	4	5
A8	I love making daily to-do lists.	1	2	3	4	5
A9	I try to organize my schedule well when I get a lot of work.	1	2	3	4	5
A10	I like to reward myself when I succeed.	1	2	3	4	5
**Practice**						
P1	I manage my time by making a schedule of daily activities.	1	2	3	4	

P2	I ask others for help in solving problems.	1	2	3	4	
P3	I do my favorite activities when dealing with stress.	1	2	3	4	
P4	I immediately lend a helping hand when my friend is in trouble.	1	2	3	4	
P5	I use a polite tone when talking to someone.	1	2	3	4	
P6	I ask for prayers from God when I am in trouble.	1	2	3	4	
P7	I express my problems to my close friends or family.	1	2	3	4	
P8	I see a counsellor when faced with problems.	1	2	3	4	
P9	I do deep breathe techniques to calm myself down.	1	2	3	4	
P10	I investigate first when I receive some news.	1	2	3	4	
