# Supplementary material for: Development of knowledge, attitude and practice questionnaires on e-Huffaz ProHealth, a multicomponent lifestyle intervention module among Tahfiz students
Source: PLoS One. 2024 Sep 26;19(9):e0309942. doi: 10.1371/journal.pone.0309942 (PMC11426467; doi:10.1371/journal.pone.0309942)
Supplement: S2 File — (PDF) [file pone.0309942.s002.pdf]

ID	Gender	Age	Status	Location	KFP1	KFP2	KFP3	KFP4	KFP5
P001	1	22	2	1	1	1	1	0	1
P002	1	22	2	1	1	0	1	1	1
P003	1	23	2	1	1	0	1	1	0
P004	1	27	2	1	1	0	1	1	1
P005	1	99	2	1	1	1	1	0	0
P006	2	40	2	2	1	1	1	1	1
P007	2	27	2	2	1	1	1	0	1
P008	2	28	2	2	1	1	1	1	1
P009	2	36	2	2	1	1	1	1	1
P010	2	25	2	2	1	1	1	1	1
P011	1	40	2	2	1	1	1	1	1
P012	1	13	1	1	1	0	1	0	1
P013	1	13	1	1	1	0	1	0	0
P014	1	13	1	1	1	0	1	1	0
P015	1	13	1	1	1	0	1	0	0
P016	1	13	1	1	1	0	1	0	1
P017	1	13	1	1	1	0	1	0	0
P018	1	14	1	1	1	0	1	1	1
P019	1	14	1	1	1	0	1	0	0
P020	1	14	1	1	1	0	1	1	1
P021	1	14	1	1	1	0	1	1	1
P022	1	15	1	1	0	0	1	0	0
P023	1	16	1	1	1	0	1	1	0
P024	2	16	1	2	1	1	1	0	1
P025	2	17	1	2	1	1	1	1	1
P026	2	17	1	2	1	1	1	0	1
P027	2	17	1	2	1	1	1	1	1
P028	2	16	1	2	1	1	1	0	1
P029	2	17	1	2	1	0	0	1	1
P030	2	17	1	2	1	0	0	1	1
P031	2	17	1	2	1	1	0	0	0
P032	2	17	1	2	1	1	1	1	1
P033	2	17	1	2	1	1	1	1	1
P034	2	17	1	2	1	0	1	1	1
P035	2	17	1	2	1	1	1	1	1
P036	2	14	1	2	1	0	1	0	1
P037	2	17	1	2	1	0	1	0	0
P038	2	17	1	2	1	0	1	1	0
P039	2	17	1	2	1	0	1	0	0
P040	1	17	1	2	1	0	0	0	0
P041	1	15	1	2	1	1	1	#NULL!	1

KFP6	KFP7	KFP8	KFP9	KFP10	KFS1	KFS2	KFS3	KFS4	KFS5
1	1	1	0	1	5	5	5	3	4
1	1	1	1	1	5	5	5	3	3
1	1	1	1	0	5	4	4	5	4
1	1	1	1	1	5	5	5	5	5
1	1	1	1	1	5	4	4	4	4
1	1	0	0	1	5	5	5	5	5
1	1	0	0	1	5	5	5	5	5
1	1	0	0	1	5	5	5	5	5
1	1	0	0	1	4	4	5	4	5
1	1	0	0	1	5	5	5	5	5
1	1	1	1	1	5	5	5	5	5
0	0	1	0	1	5	4	5	3	4
1	0	1	1	1	5	5	4	2	2
1	0	0	0	1	5	5	4	4	3
1	0	1	1	0	4	5	3	3	4
1	0	1	0	0	5	4	4	4	3
1	0	0	1	1	5	4	3	5	2
1	0	1	0	1	5	5	5	5	4
1	0	1	1	1	5	5	4	5	3
0	1	1	1	0	5	4	5	4	3
1	0	0	0	1	5	5	4	4	4
0	0	1	1	1	5	5	4	4	3
1	0	1	0	1	5	5	4	4	3
1	1	0	0	1	5	5	4	5	5
1	1	0	0	1	4	4	4	5	4
1	1	0	0	1	5	5	3	4	3
1	1	0	0	1	5	5	5	5	5
1	1	0	0	1	5	4	4	5	1
1	1	0	0	1	5	3	5	5	5
1	0	0	0	1	4	3	4	4	2
1	1	0	0	1	5	4	4	4	5
1	1	0	0	0	5	5	5	5	5
1	1	0	0	1	5	5	5	5	5
1	0	0	0	1	5	5	5	5	3
1	1	0	0	1	1	1	1	1	3
1	1	0	0	1	5	4	4	5	4
1	1	0	0	0	5	4	4	3	4
1	1	0	0	1	5	4	4	3	5
0	1	0	0	1	4	4	4	3	4
1	1	0	0	1	5	5	5	4	5
1	1	0	0	1	5	5	5	5	5

KFS6	KFS7	KFS8	KFS9	KFS10	KFA1	KFA2	KFA3	KFA4	KFA5
4	5	3	5	5	3	4	2	3	3
5	5	5	5	5	4	4	3	3	3
4	5	4	5	5	4	4	2	3	3
5	5	5	5	5	4	4	4	4	4
3	4	5	4	3	4	4	2	4	3
5	5	5	5	5	4	4	4	4	3
5	5	5	5	5	4	4	4	3	2
5	5	5	5	5	3	4	3	4	3
4	5	4	5	5	4	4	2	3	3
5	5	5	5	3	4	4	3	4	4
5	5	5	5	5	4	4	4	4	3
3	3	3	4	3	3	4	3	2	1
4	5	4	4	5	4	4	1	3	3
3	4	3	5	5	3	4	2	3	2
5	5	5	5	5	3	4	3	3	2
4	4	3	4	5	3	3	4	4	3
1	5	3	5	4	3	4	1	2	2
5	5	3	5	5	4	4	3	4	4
5	5	4	5	4	3	4	1	4	4
2	5	4	5	5	3	4	2	4	3
5	5	4	4	4	3	2	3	2	3
3	4	4	5	4	4	4	2	3	3
4	4	3	5	5	4	4	3	3	3
5	5	5	5	5	4	4	3	3	4
5	5	4	4	4	4	4	4	3	2
4	4	3	4	4	4	4	3	3	2
5	5	5	4	5	4	4	3	3	2
4	4	4	4	2	4	4	3	4	2
5	5	5	5	3	4	4	3	3	3
4	4	4	4	4	4	4	3	2	2
5	5	4	5	5	4	4	3	2	2
5	5	5	5	5	4	4	3	2	2
5	5	5	5	5	4	4	3	4	3
5	5	5	5	5	4	4	2	3	3
1	1	1	2	1	4	4	2	4	2
5	5	4	3	4	4	3	2	4	2
4	5	4	5	4	4	4	2	2	2
4	5	4	3	4	4	4	2	3	2
3	4	3	4	4	4	3	2	2	1
5	5	4	5	5	3	3	1	4	1
5	5	5	5	5	4	4	4	3	3

KFA6	KFA7	KFA8	KFA9	KFA10	PP1	PP2	PP3	PP4	PP5
4	2	4	3	4	1	0	1	0	1
3	3	4	4	4	1	1	1	1	1
3	3	4	3	4	1	1	1	0	0
4	4	4	4	4	1	1	1	1	1
2	2	3	2	4	1	1	1	1	1
3	3	4	3	4	1	1	1	1	1
1	3	4	1	4	1	1	1	1	0
3	3	4	3	4	1	1	1	1	1
3	3	4	2	4	1	1	0	1	1
1	1	4	4	4	1	1	1	1	1
3	3	4	3	4	1	1	1	1	1
1	2	3	1	2	0	0	0	0	0
4	2	4	4	4	1	1	0	1	1
3	1	4	3	4	1	1	1	1	1
3	3	4	3	4	1	0	1	0	1
3	4	4	3	4	1	1	1	1	1
1	1	4	2	4	1	1	1	0	0
1	3	4	1	4	1	1	1	0	1
2	2	4	4	4	1	0	0	0	0
4	2	4	3	4	1	1	0	0	1
2	2	2	3	4	1	0	0	1	0
2	3	4	4	4	0	1	0	1	1
1	3	4	3	4	1	1	1	0	0
2	2	4	2	4	1	1	1	1	1
2	2	3	1	3	1	1	1	1	1
1	2	4	1	4	1	1	1	1	0
2	4	4	3	4	1	1	1	1	1
2	3	4	3	4	1	1	1	1	1
2	2	4	2	4	1	1	1	1	1
2	2	4	3	4	1	0	1	1	1
1	4	4	1	4	1	1	1	1	1
2	2	4	2	4	1	1	1	1	1
2	3	4	2	4	1	1	1	1	0
2	3	4	3	4	1	1	1	1	0
1	3	4	2	4	1	1	1	1	1
2	3	4	3	4	1	1	1	1	0
1	3	4	2	4	0	1	0	0	0
2	2	3	3	3	1	1	1	1	0
1	2	3	3	3	1	1	0	0	0
1	2	3	1	3	1	1	1	0	0
2	3	4	2	4	1	1	1	1	1

PP6	PP7	PP8	PP9	PS1	PS2	PS3	PS4	PS5	PS6
1	0	0	1	3	4	4	4	3	3
1	0	0	1	4	4	4	4	4	2
1	1	0	1	4	5	5	4	4	3
1	0	0	1	5	5	5	5	5	5
1	1	1	1	3	3	5	2	3	2
1	1	0	1	4	5	5	5	5	2
1	1	1	1	4	4	5	4	4	1
1	1	1	1	4	4	4	5	4	1
1	1	1	1	4	5	5	5	5	1
1	1	1	1	5	5	5	5	5	1
1	0	0	1	5	5	5	5	5	2
0	0	0	1	3	3	5	3	3	3
1	0	1	1	2	2	5	4	2	1
1	1	0	1	5	5	5	5	3	3
0	0	1	1	3	4	5	3	4	2
0	0	0	1	5	4	4	4	4	2
1	0	1	0	5	5	5	4	5	2
1	0	0	1	2	5	5	5	5	2
1	1	1	1	5	4	5	4	3	2
0	0	1	1	5	5	5	4	4	3
0	0	0	1	4	4	5	4	4	3
0	1	0	1	3	5	5	3	3	2
0	0	0	1	4	4	5	3	3	3
1	1	1	1	4	4	5	5	5	2
1	0	1	1	3	4	5	4	3	2
1	0	1	1	3	4	4	5	3	1
1	1	1	1	4	4	5	4	4	3
1	0	1	1	4	4	5	4	5	1
1	0	0	1	3	2	5	3	2	3
1	0	0	1	4	5	5	5	2	2
1	1	1	1	4	5	5	5	4	1
1	0	0	1	5	5	5	5	5	5
1	0	1	1	4	5	5	5	5	2
1	0	1	1	5	5	5	5	5	2
1	0	1	1	3	5	5	3	3	3
0	1	0	1	3	4	5	3	3	1
1	1	1	1	3	3	4	3	3	2
1	0	0	1	3	3	4	3	3	4
0	0	0	1	4	3	5	4	3	2
1	0	0	1	4	4	4	3	4	3
1	0	1	1	5	5	5	5	5	4

PS7	PS8	PS9	PA1	PA2	PA3	PA4	PA5	PA6	PA7
2	1	3	2	2	4	1	2	3	1
3	3	5	3	3	3	3	3	4	3
4	3	4	2	4	3	3	3	3	3
5	5	5	4	3	4	4	4	4	4
3	4	5	2	2	4	2	3	3	1
4	5	5	3	3	4	2	3	4	4
3	3	5	3	3	3	3	4	4	3
4	3	5	3	4	4	3	4	4	3
4	5	5	3	4	4	1	2	4	4
5	5	5	4	4	4	4	4	4	1
3	4	5	3	3	4	4	3	4	3
3	3	4	1	1	3	1	3	4	1
1	1	2	2	2	2	1	2	4	2
5	5	5	1	2	3	2	1	4	3
3	3	5	2	2	3	2	3	4	2
4	3	5	3	3	4	3	1	4	3
2	4	4	3	2	2	3	2	4	2
5	3	5	4	4	4	4	4	4	4
3	1	4	2	3	4	2	1	4	2
5	5	5	4	3	4	2	3	4	3
4	4	5	3	3	4	2	3	3	3
3	3	5	2	3	4	3	2	3	3
4	5	5	3	3	4	3	3	4	4
4	4	4	2	3	4	2	3	4	4
3	3	4	3	2	3	2	4	4	3
5	3	4	2	2	4	2	4	2	2
3	3	4	3	3	4	4	4	4	4
4	4	5	2	3	4	3	1	4	4
1	3	5	2	2	4	2	4	4	3
3	3	4	2	3	2	1	2	3	2
5	3	4	3	2	4	1	4	4	4
5	5	5	2	2	3	3	3	3	3
4	4	5	3	3	4	2	1	4	3
5	5	5	3	3	4	3	4	4	3
4	3	4	2	1	3	2	4	4	1
3	5	5	2	2	4	3	2	4	4
3	3	4	2	2	3	2	1	2	2
3	3	4	2	1	2	2	3	3	2
4	3	4	2	2	2	2	3	3	2
3	3	3	1	2	4	1	2	2	2
4	4	5	3	3	4	3	4	4	3

PA8	PA9	KPP1	KPP2	KPP3	KPP4	KPP5	KPP6	KPP7	KPP8
4	2	1	1	1	1	1	0	1	1
4	3	1	1	1	1	1	1	1	1
1	3	1	0	0	0	1	0	1	1
4	4	1	1	1	1	1	0	1	1
4	2	1	1	1	0	1	1	1	0
2	3	1	1	1	1	0	0	1	1
2	3	1	1	1	1	1	0	1	1
1	3	1	1	1	1	1	0	1	1
1	4	1	1	1	1	1	0	1	1
1	4	1	1	1	1	0	0	1	1
2	4	1	1	1	1	0	0	1	1
3	2	0	1	1	1	0	0	0	0
4	4	0	0	1	0	1	0	1	0
4	2	1	0	0	0	0	0	1	1
4	3	1	1	0	1	1	0	1	1
3	3	1	0	1	1	1	0	1	1
4	4	1	1	0	0	0	0	1	1
4	4	1	1	1	1	0	0	1	1
2	3	1	0	0	1	0	1	1	1
5	1	1	0	1	1	0	1	1	1
4	4	1	1	1	1	0	0	1	0
2	2	1	0	0	0	1	0	1	1
3	3	1	0	0	1	0	0	1	1
2	2	1	1	1	1	0	0	1	1
3	3	1	1	1	1	0	0	1	1
2	1	1	1	1	1	0	0	1	1
1	3	1	1	1	1	0	0	1	1
1	2	1	0	1	1	1	0	1	1
3	2	1	1	1	0	0	0	1	0
3	2	1	1	1	1	1	0	1	1
2	4	1	1	1	1	1	0	1	1
4	3	1	1	1	1	0	0	1	1
3	2	1	1	1	0	1	0	1	1
3	4	1	1	1	1	1	0	1	1
4	2	1	1	1	1	0	0	1	1
3	3	1	1	1	1	1	0	1	0
1	3	1	1	1	1	1	0	1	1
4	2	1	1	1	0	1	0	1	1
3	2	0	0	1	0	0	1	1	1
4	2	1	0	1	0	0	0	1	1
2	3	1	1	1	1	1	0	1	1

KPP9	KPP10	KPS1	KPS2	KPS3	KPS4	KPS5	KPS6	KPS7	KPS8
1	1	4	5	2	4	4	5	1	3
1	1	4	5	3	4	3	3	1	3
1	1	5	5	3	5	5	4	1	3
1	1	5	5	1	5	5	5	1	5
1	1	4	5	2	3	3	4	1	3
1	1	5	5	2	4	5	4	1	4
1	1	4	4	2	4	3	3	1	3
1	1	4	4	1	4	5	4	1	4
1	1	5	5	2	5	4	3	1	4
1	1	5	5	1	5	5	3	1	5
1	1	5	5	5	5	5	5	1	3
0	0	3	5	3	3	3	4	5	3
1	0	4	4	2	3	4	4	2	2
1	1	4	5	4	5	5	5	3	3
1	1	4	3	1	5	4	4	1	1
1	1	4	5	2	5	5	4	2	4
1	1	5	5	4	4	5	5	2	4
1	1	5	4	2	5	4	5	4	2
1	1	4	4	5	5	2	1	1	4
1	1	4	5	3	5	5	5	1	4
1	0	5	4	4	4	5	4	4	4
1	0	4	5	3	4	4	5	1	3
1	1	5	4	3	5	5	5	3	5
1	1	4	5	2	4	3	2	1	3
1	1	4	4	4	4	5	3	1	4
1	1	3	5	3	4	4	3	3	3
1	1	5	5	5	5	5	3	2	5
1	1	5	5	1	5	5	1	1	5
1	1	5	5	3	4	5	3	1	4
1	1	4	4	2	3	3	3	1	3
1	1	4	4	3	3	5	4	1	4
1	1	5	5	2	5	5	2	2	2
1	1	4	4	2	4	4	3	1	4
1	1	5	5	2	4	4	2	2	4
1	1	2	5	1	3	3	5	5	3
1	1	3	5	1	5	4	5	1	4
1	1	4	4	2	4	4	3	1	3
1	1	5	4	3	3	3	4	1	3
1	0	5	5	1	4	4	3	1	4
1	1	3	3	4	3	3	3	2	3
1	1	5	5	2	4	5	4	1	3

KPS9	KPS10	KPA1	KPA2	KPA3	KPA4	KPA5	KPA6	KPA7	KPA8
3	4	1	1	3	2	3	4	1	1
3	4	3	3	3	3	3	4	2	1
5	4	3	3	3	4	4	4	4	2
5	5	4	3	4	4	4	4	2	2
4	5	3	2	4	3	3	4	2	1
5	5	4	4	4	4	4	4	4	3
4	3	4	3	2	3	4	4	2	1
4	5	4	1	3	4	4	4	3	1
4	5	4	3	4	4	4	4	2	1
5	5	4	1	4	4	4	4	4	1
5	4	3	3	3	3	4	4	4	2
3	4	1	1	4	3	3	3	1	1
2	1	3	2	1	3	3	4	1	1
4	5	1	3	3	4	4	4	3	1
4	3	2	3	4	4	2	4	2	1
4	4	3	2	4	3	4	4	3	2
4	3	3	4	4	4	4	4	4	3
2	2	2	4	4	4	3	4	4	4
4	5	3	1	4	2	2	2	3	1
5	5	3	4	4	4	3	4	4	1
5	5	3	3	4	3	4	4	4	3
5	3	2	2	3	3	4	4	1	1
5	5	4	4	3	4	4	4	3	3
5	5	3	3	4	4	3	4	3	3
4	4	4	4	3	3	3	4	4	3
4	4	2	2	2	2	2	4	2	2
5	4	4	3	2	4	3	4	4	2
5	5	4	4	4	4	4	4	4	4
4	5	4	3	4	4	3	4	4	1
4	4	2	1	3	3	1	4	2	1
3	3	3	3	2	3	4	4	2	1
5	5	4	4	4	4	4	4	2	2
4	4	3	3	3	3	4	4	3	3
4	4	3	3	3	3	4	4	3	3
5	2	2	1	1	1	2	4	1	1
5	4	3	2	2	3	4	4	2	3
5	1	4	2	3	3	3	4	2	1
3	3	2	3	3	3	3	4	3	3
5	4	3	2	3	3	3	4	3	1
4	3	2	2	2	2	2	3	3	1
5	5	4	4	4	3	4	4	4	4

KPA9	KPA10
3	2
2	4
3	3
4	4
2	5
3	3
3	3
4	4
4	4
4	4
2	4
3	3
2	3
2	3
2	4
3	3
2	3
4	4
1	3
4	3
3	3
2	2
4	4
4	3
4	3
2	2
4	4
4	4
4	4
3	3
4	4
2	3
3	4
4	4
1	3
4	4
3	4
2	3
2	3
2	2
4	3
